# Transcriptome sequencing and profiling of expressed genes in cambial zone and differentiating xylem of Japanese cedar (*Cryptomeria japonica*)

**DOI:** 10.1186/1471-2164-15-219

**Published:** 2014-03-20

**Authors:** Kentaro Mishima, Takeshi Fujiwara, Taiichi Iki, Katsushi Kuroda, Kana Yamashita, Miho Tamura, Yoshitake Fujisawa, Atsushi Watanabe

**Affiliations:** 1 Forest Tree Breeding Center, Forestry and Forest Products Research Institute, 3809-1 Ishi, Juo, Hitachi, Ibaraki 319-1301, Japan; 2 Forestry and Forest Products Research Institute, 1 Matsunosato, Tsukuba, Ibaraki 305-8687, Japan; 3 Department of Forest Environmental Sciences, Faculty of Agriculture, Kyushu University, 6-10-1 Hakozaki, Higashi-ku, Fukuoka 812-8581, Japan

**Keywords:** *Cryptomeria japonica*, cDNA library, Microarray, Cambium

## Abstract

**Background:**

Forest trees have ecological and economic importance, and Japanese cedar has highly valued wood attributes. Thus, studies of molecular aspects of wood formation offer practical information that may be used for screening and forward genetics approaches to improving wood quality.

**Results:**

After identifying expressed sequence tags in Japanese cedar tissue undergoing xylogenesis, we designed a custom cDNA microarray to compare expression of highly regulated genes throughout a growing season. This led to identification of candidate genes involved both in wood formation and later cessation of growth and dormancy. Based on homology to orthologous protein groups, the genes were assigned to functional classes. A high proportion of sequences fell into functional classes related to posttranscriptional modification and signal transduction, while transcription factors and genes involved in the metabolism of sugars, cell-wall synthesis and lignification, and cold hardiness were among other classes of genes identified as having a potential role in xylem formation and seasonal wood formation.

**Conclusions:**

We obtained 55,051 unique sequences by next-generation sequencing of a cDNA library prepared from cambial meristem and derivative cells. Previous studies on conifers have identified unique sequences expressed in developing xylem, but this is the first comprehensive study utilizing a collection of expressed sequence tags for expression studies related to xylem formation in Japanese cedar, which belongs to a different lineage than the Pinaceae. Our characterization of these sequences should allow comparative studies of genome evolution and functional genetics of wood species.

## Background

Wood represents the main source for terrestrial biomass production and is a major renewable resource for the timber, paper, and bioenergy industries [1]. Genomics approaches have been applied to explore the molecular basis of growth and development in a few forest tree species with economic relevance. Transcript profiling in trees has also specifically focused on wood formation (xylogenesis) because of the ecological significance of forest trees and the economic importance of wood [2-4]. Wood formation begins from the cambium and generates wood as the end product of secondary vascular system development, which proceeds from cell division to expansion, secondary wall formation, lignification, and finally programmed cell death [5,6]. Notably, identification of accumulated expressed sequence tags (ESTs) and their expression pattern during wood formation has been achieved in target species for breeding, such as *Pinus*, *Populus* and *Picea*[1,2,6-14].

Japanese cedar (*Cryptomeria japonica*) is an allogamous coniferous species that relies on wind-mediated pollen and seed dispersal, and it is one of the most important forestry tree species in Japan. The Japanese cedar tree has excellent attributes (straight bole, rapid growth, ease of processing, and pleasant color and scent), and it has been used for house construction, to build wooden ships, barrels, and musical instruments, and for many products intended for daily use for hundreds of years [15]. More than 3,700 Japanese cedar trees have been planted throughout Japan, covering an area of 4.5 million ha and accounting for 44% of Japan’s artificial forests. Seventeen million seedlings are supplied as planting material for forestation every year, making this species very important for Japanese forestry today, as it has been since ancient times [16].

Next-generation sequencing can be a more efficient approach for obtaining functional genomic information. This type of sequencing can result in high transcriptome coverage depth and facilitates the *de novo* assembly of transcriptomes from species where full genomes do not exist [17,18]. In addition, by simultaneously measuring the abundance of transcripts for thousands of genes with accumulated sequence information, microarray analysis promises a comprehensive understanding of regulatory gene functions and the growth and development of plants [19]. To understand the molecular mechanisms involved in wood formation and key targets for genetic manipulation and selection of superior wood quality, these techniques will be powerful and efficient tools [20]. The only molecular studies of wood formation in Japanese cedar have identified large numbers of genes that are expressed in male strobili [21]. However, very limited genomics and functional genomics resources related to wood formation are publicly available for Japanese cedar.

The first objective of this paper was to produce an extensive collection of sequenced ESTs found in xylem and cDNA clones to support manufacture of cDNA microarrays and gene discovery efforts in Japanese cedar. The next goal was to elucidate a comprehensive expression profile in the growing season using these microarrays. For this purpose, we identified 55,051 unique sequences by next-generation Roche 454 sequencing using a non-normalized cDNA library from the cambial meristem and its derivatives from Japanese cedar. To gain further insight into seasonal expression patterns, a custom cDNA microarray was designed from the cDNA library obtained and from EST data (inner bark data on ForestGen; http://forestgen.ffpri.affrc.go.jp) [22] and was used to investigate differential gene expression in Japanese cedar during wood formation.

## Results and discussion

### Microscopic observation of differentiating xylem

**Figure 1:**
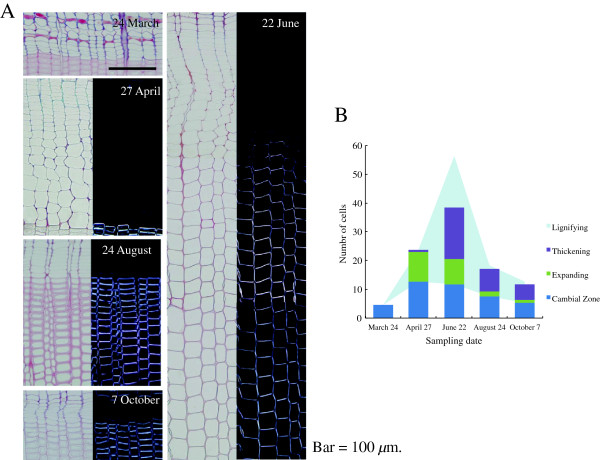
**Cross-sections of cambial zone and differentiating xylem of *Cryptomeria japonica *trees. A**. Cross-sections viewed under an ordinary light microscope (24 March) and pairs of ordinary (left) and polarizing (right) light microscope images for the same field (27 April, 22 June, 24 August and 7 October). **B**. Number of cells in cambial zone and differentiating xylem. Cells in differentiating xylem were categorized into expanding cells, thickening cells and lignifying cells in accordance with ordinary and polarizing light microscope observations.

Based on anatomical observation of the cambial zone and the differentiating xylem, the tissue underwent seasonal cycles in activity of xylem formation, including cell division, secondary wall formation and lignification, through the growing season (Figure 1A,B). The cambial cells were not active in samples taken on 24 March. An average of only 4.8 cambial cells was found in each radial file, significantly fewer than found in other samples collected in April (*p* < 0.01), June (*p* < 0.01) and August (*p* < 0.05). The expanding tracheids occupied the most space in differentiating xylem in samples taken on 27 April. The number of these tracheids was larger on this date than in samples collected during the growing season. Thus, formation of derived tracheids was most active in samples collected on 27 April. A few secondary wall-forming tracheids were found in some radial files. This indicated that secondary wall formation might have just been reactivated around the day of sampling. The largest number of tracheids at the stage of secondary wall formation and lignification was observed in the 22 June samples. The number was significantly larger than in the other samples collected (*p* < 0.01). Therefore, the peak activity in xylem formation, including cell differentiation and secondary wall formation, was found in the 22 June samples.

The number of expanding tracheids in each radial file had significantly decreased from an average of 8.8 cells in samples taken on 22 June to an average of 1.7 cells in samples taken on 24 August (*p* < 0.01). This indicated that cell division activity in the cambial zone was lower than at earlier stages. Thus, the major activities in differentiating xylem that could be observed microscopically were secondary wall formation and lignification in the samples collected in August and October.

### EST sequencing and *de novo* assembly

**Figure 2:**
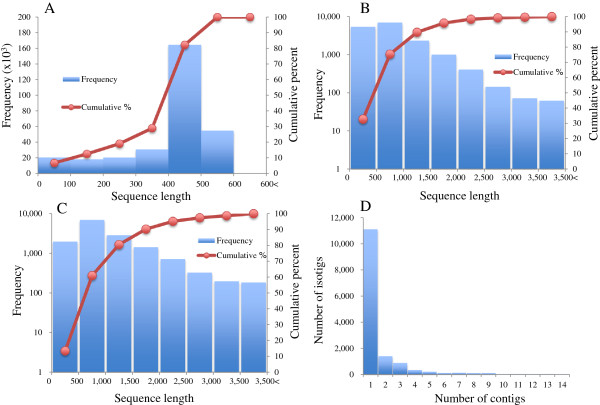
**Summary of EST sequencing and *de novo *assembly. A)** Size distribution and cumulative percentage of raw sequence reads. **B)** Size distribution and cumulative percentage of contigs. **C)** Size distribution and cumulative percentage of isotigs. **D)** Distribution of isotig sequence coverage.

Sequencing of cDNA libraries generated a total 308,542 raw reads, with an average length of 405.29 bp. The size distribution of raw reads is shown in Figure 2A, and a summary of sequencing and assembly results is presented in Table 1. After trimming the adaptors and primer sequences, 9,764 sequences were removed due to short length, low complexity, or overall low quality scores. This cleaning and trimming step resulted in 298,778 high-quality reads, corresponding to 96.8% of the original raw sequence. A total of 241,696 high-quality reads was assembled into 11,022 contiguous sequences (contigs over 500 bp), and 40,435 reads were identified as singletons (i.e., reads not assembled into contigs). The size of contigs ranged from 100 to 9,656 bp, with an average length of 1,014 bp. The distribution of contig size is shown in Figure 2B. Contiguous sequences were further assembled into 14,616 isotigs. Isotigs are putative transcripts constructed using the overlapping contig reads provided as input to Newbler cDNA assembler. The size distribution of isotigs ranged from 33 to over 9,656 bp, with an average length of 1,069 bp (Figure 2C). More than 99% of the isotigs were over 100 bp and 50% of the assembled bases were incorporated into isotigs longer than 1,261 bp (N50 = 1,261 bp). The coverage depth for isotigs ranged from 1 to 14, with an average of 1.7 contigs assembled into each isotig (Figure 2D). The isotigs and singletons together resulted in 55,051 unique sequences (Additional file 1: Figure S1).

**Table 1:** ***C. japonica *** **transcriptome sequencing and assembly summary**

	**Sequence**	**Bases (Mbp)**
Sequencing		
Raw sequencing reads	308,542	125.1
Average read length	405.29 bp	
Assembly		
Trashed	9764	
Reads used in assembly	298,778	121.2
Average read length	405.69 bp	
Contigs		
All contigs		
Reads assembled as contigs	241,696	98.6
Number of contigs (over 100 bp)	15,521	12.7
large contigs (over 500 bp)		
Number of contigs	11,022	11.2
Average contigs size	1,014	
Largest contigs length	9,656	
N50 contig size	1,102	
Isotigs		
Number of isotigs	14,616	15.6
Average isotigs size	1,069	
Largest isotigs length	9,656	
N50 isotig size	1,261	
Avrage contig count	1.7	
Singletons	40,435	
Unique sequences	55,051	

### Sequence comparison with other species

**Figure 3:**
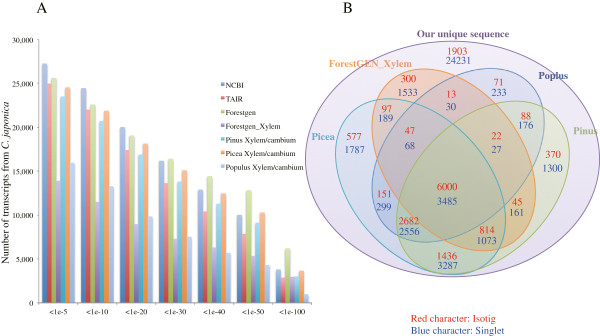
**Sequence similarities. A)** Number of transcript sequences from *C. japonica* cambium region similar to sequences in the NCBI, TAIR, ForestGen, ForestGen_Xylem, pine, spruce, and poplar databases according to BLASTx and tBLASTx E-value cutoff values. **B)** Venn diagram showing the overlap between our collected unique sequences and sequences in four other databases according to a tBLASTx search (*E*-value <1e-5).

All unique sequences were searched against the sequences in the National Center for Biotechnology Information (NCBI) non-redundant protein database and The Arabidopsis Information Resource (TAIR) using a BLASTx algorithm *E-*value of 1e-5 (Figure 3A). A total of 12,606 isotigs (86.2% of all isotigs) and 14,688 singleton sequences (36.3% of all singletons) had significant BLAST matches at NCBI, and 11,958 isotigs (81.8%) and 13,027 singletons (32.2%) had significant BLAST matches at TAIR. When we compared our unique sequences with EST sequences in the Japanese cedar database (ForestGen) and libraries including xylem and cambium tissue from The Gene Index and ForestGen_Xylem (inner bark and sapwood) using a tBLASTx algorithm with an *E-*value of 1e-5, we found that 25,641 (11,278 isotigs and 14,363 singletons) had significant BLAST matches at ForestGen and 23,524 (11,457 isotigs and 12,067 singletons) to transcripts from *Pinus*, 24,550 (11,804 isotigs and 12,746 singletons) from *Picea*, and 15,945 (9,074 isotigs and 6,871 singletons) from *Populus* and that 13,906 (7,338 isotigs and 6,568 singletons) sequences from ForestGen_Xylem included similar ESTs. The largest overlap was found for the ForestGen database based on lower E-values. A larger overlap was found for other coniferous species than for broadleaf species. Comparison with BLAST results against the ForestGen and ForestGen_Xylem databases indicated that the unique sequences collected in this study were also covered by ESTs previously collected from other organs of Japanese cedar (Figure 3A). When comparing EST sequences in other libraries involving xylem and cambium, though the smallest overlap was found for ForestGen_Xylem, most of the unique sequences overlapped those of three well-known species undergoing xylogenesis (Figure 3B). These results clarified that the previously accumulated Japanese cedar xylogenesis related ESTs were incomplete, whereas our data mostly coincided with ESTs collected from xylem and cambium in other species.

Therefore, our data are expected to be a useful resource for ESTs related to xylem or cambium development in Japanese cedar.

### Identifying protein families represented in sequences by Pfam

The unique sequences were investigated for conserved domains using the Pfam database [23] to predict their function. In 55,051 unique sequences, we found that 19,887 (36.1%) of the encoded proteins were similar to members of 4,764 Pfam protein families (*E*-value < 1e-10). Overall, products of 18,915 (34.4%) of the transcripts from Japanese cedar cambium tissue were similar to members of 4,420 Pfam families when domains of unknown function (DUFs) (317 families) and uncharacterized protein families (UPFs) (27 families) were excluded. The 20 most abundant protein families in cambium tissue of Japanese cedar are shown in Table 2. The frequency of occurrence of members of these families corresponded with previous reports on Japanese cedar male strobili and white spruce [21,24].

**Table 2:** **Occurrence of the 20 most common Pfam domains in the predicted proteins of unique transcripts from cambium and differentiating xylem of *C. japonica***

**Description of Pfam domain**	**Number of *C. japonic*** **a transcripts**^**a**^	**Pfam accession**
Protein kinase domain	563	PF00069
NB-ARC domain	486	PF00931
Leucine-rich repeat	197	PF13855 (including PF07714)
Tyrosine kinase	174	PF07714
RNA recognition motif	173	PF00076
Cytochrome P450	171	PF00067
PPR repeat family	129	PF13041
UDP-glucoronosyl and UDP-glucosyl transferase	113	PF00201
Reverse transcriptase (RNA-dependent DNA polymerase)	110	PF00078 (including PF07727)
WD domain, G-beta repeat	102	PF00400
TIR domain	98	PF01582
DEAD/DEAH box helicase	91	PF00270
Alpha/beta hydrolase fold	82	PF12697
AAA proteins	80	PF00004
RING finger domain	78	PF13639
ATP-binding domain of ABC transporters	77	PF00005
Sugar (and other) transporter	72	PF00083
SET domain	67	PF00856
Mitochondrial carrier protein	65	PF00153
Protein phosphatase 2C	65	PF00481

^a^Protein families were identified by BlastX searches with *E*-value <1e-10 in the Pfam database.

### Identifying proteins according to clusters of orthologous groups (COGs) from seven eukaryotic genomes represented in sequences

**Figure 4:**
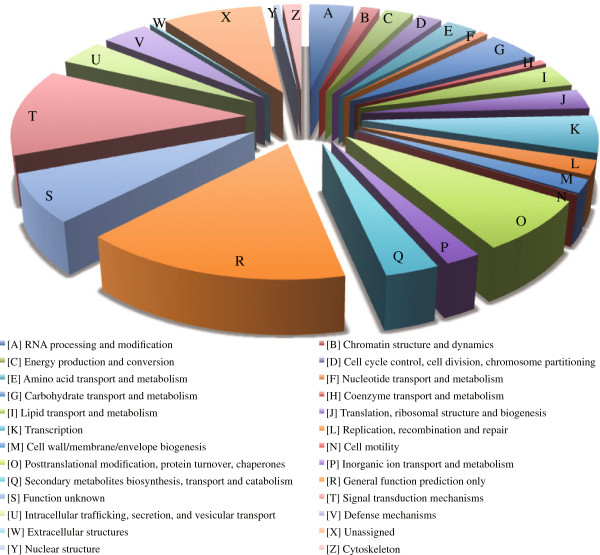
**Functional classification and relative levels of ESTs derived from cambium region of *C. japonica.*** Values are shown as percentage of unique transcripts in the pool.

The unique sequences were searched against the COG database [25] using the BLASTx program. The sequences that showed significant similarity (an *E*-value < 1e-5) with those in the database were annotated and assigned to designated functional classes. Overall, 22,738 sequences (41.3%) were annotated to known sequences with designated functional classifications, and 3,816 were similar to known genes of unknown function and unassigned sequences in the database (Figure 4). The most frequent functional categories for our data were “Posttranslational modification, protein turnover, chaperones (category symbol O)” and “general function prediction only (R),” which agreed with previous reports [21,26-28]. On the other hand, “signal transduction mechanisms (T)” was the next largest category in the annotated designations of functional classification, including the function unknown (and unassigned) category. This feature differed from previous reports, suggesting that seasonal expression of genes specific to cambial region tissues occurs.

### Identification of transcription factors

Transcription factors are proteins that function in controlling the expression of target genes quantitatively, temporally, and spatially [29]. The unique sequences we identified were annotated against the PlnTFDB [30], a recently developed database of transcription factor families for 22 plant species, using the BLASTx program. BLASTx searches revealed 3,085 unique sequences of Japanese cedar with matches against *Arabidopsis thaliana* and 2,735 unique sequences with matches against *Populus trichocarpa* with *E*-values < 1e-5. Of the 82 transcription factor families, these sequences were annotated to 79 in the *Arabidopsis* genome and 77 in the poplar genome (Tables 3 and 4). The most abundant transcription factor family annotated to *A. thaliana* was *WRKY* (*WRKY DNA-binding domain*), with 218 unique sequences, whereas for *P. trichocarpa*, it was *C3H* (*Cys4-His-Cys3 zinc finger*), with 158 unique sequences. In particular, unique sequences for transcription factors associated with xylogenesis (such as the MYB, NAC and HB transcription factors) were abundant. Therefore, in future studies, it will be necessary to specify which family members are associated with xylogenesis in this species. These transcription factor features were similar to those reported for radiata pine and white spruce [3,24].

**Table 3:** **Identification of transcripts encoding putative transcription factors in cambium and differentiating xylem in *C. japonica *** **against *Populus trichocarpa***

**TF family**	**Description**	**vs Populus**	
		**ESTs**	%^*^
C3H	Zinc finger, C-x8-C-x5-C-x3-H type	160	5.82
NAC	No apical meristem (NAM) domain	157	5.71
PHD	Cys4--His--Cys3 zinc finger	146	5.31
AP2-EREB	PAP2 domain	139	5.06
HB	Homeobox domain	126	4.58
bHLH	Helix-loop-helix DNA-binding domain	123	4.47
SNF2	ATP binding/DNA binding/helicase/nucleic acid binding	116	4.22
WRKY	WRKY DNA-binding domain	115	4.18
C2H2	Zinc finger, C2H2 type	113	4.11
MYB	Myb-like DNA-binding domain	110	4.00
Orphans	antiporter/multidrug efflux pump/transporter	107	3.89
MYB-related	N-terminal myb-domain	95	3.46
ARF	Auxin response factor	68	2.47
SET	SET domain	61	2.22
bZIP	Basic leucine zipper (bZIP) motif	59	2.15
Trihelix	Trihelix DNA-binding domain	58	2.11
CCAAT	NUCLEAR FACTOR Y, SUBUNIT A10	54	1.96
G2-like	PRENYLATED RAB ACCEPTOR 1.G2	54	1.96
GRAS	GRAS protein	54	1.96
TRAF	TRAF homology domain-containing protein	48	1.75
ABI3VP1	ABI3/VP1 protein	44	1.60
MADS	DNA-binding and dimaerization domain	41	1.49
FAR1	N-terminal microtubule binding motor domain	40	1.46
GNAT	GCN5-related N-acetyltransferase (GNAT) family protein	39	1.42
mTERF	mitochondrial transcription termination factor family protein	36	1.31
Jumonji	nucleic acid binding/zinc ion binding	31	1.13
TCP	ATP binding/protein binding	30	1.09
HSF	Heat shock factor	26	0.95
C2C2-Dof	Dof zinc finger	23	0.84
FHA	Forkhead domain	23	0.84
DBP	protein phosphatase 2C	22	0.80
CPP	copalyl pyrophosphate (CPP) of gibberellin biosynthesis	21	0.76
zf-HD	zf-HD class homeobox domain	21	0.76
LOB	LATERAL ORGAN BOUNDARIES	20	0.73
SBP	SBP domain	20	0.73
AUX/IAA	AUX/IAA family	19	0.69
C2C2-GATA	GATA zinc finger	19	0.69
ARID	AT-rich interaction domain	17	0.62
HMG	HMG (high mobility group) domain	17	0.62
PLATZ	Plant AT-rich sequnce and zinc-binding protein1	17	0.62
LUG	LEUNIG gene	16	0.58
CAMTA	Calmodulin-binding transcription activators	15	0.55
RWP-RK	RWP-RK domain-containing protein	15	0.55
ARR-B	Arabidopsis response regulator B	14	0.51
CSD	superoxide dismutase	14	0.51
Tify	JASMONATE-ZIM-DOMAIN PROTEIN 6	14	0.51
BES1	BRI1-EMS supressor	13	0.47
E2F-DP	DNA binding/protein heterodimerization	13	0.47
SWI/SNF-SWI	chromatin binding/protein binding	10	0.36
Alfin-like	Cys4 zinc finger and His/Cys3	9	0.33
C2C2-YABBY	YABBY transcription activator	9	0.33
GRF	Growth regulation factor1	9	0.33
BSD	BSD domain-containing protein	8	0.29
EIL	Ethylene insensitivel (EIN3)	8	0.29
OFP	predicted nuclear localization signal	8	0.29
Pseudo ARR-B	Pseudo Arabidopsis response regulator B	8	0.29
TUB	structural constituent of cytoskeleton	8	0.29
C2C2-CO-like	CCT motif	7	0.25
DDT	DDT domain-containing protein	7	0.25
Sigma70-like	DNA binding/DNA-directed RNA polymerase	7	0.25
SWI/SNF-BAF	SWIB complex BAF60b domain-containing protein	7	0.25
GeBPD	NA-binding storekeeper protein-related	6	0.22
LIM	LIM domain	5	0.18
TAZ	TAZ zinc finger	5	0.18
VOZ	VOZ domain	5	0.18
Coactivator p15	transcriptional coactivator p15 (PC4) family protein	3	0.11
IWS1	molecular_function unknown	3	0.11
BBR/BPC	DNA binding	2	0.07
PBF-2-like	peptidase/threonine-type endopeptidase	2	0.07
Rcd1-like	RADICAL-INDUCED CELL DEATH1	2	0.07
SRS	Domain unknown function	2	0.07
HRT	nucleotide binding	1	0.04
MED6	RNA polymerase transcriptional regulation mediator-related	1	0.04
MED7	MED7 domain	1	0.04
RB	Retinoblastoma-associated protein B domain	1	0.04
SOH1	SOH1 domain	1	0.04
ULT	DNA binding	1	0.04

^*^% is based on the 2749 Japanese cedar unique sequences with homologs in the PlnTFDB.

**Table 4:** **Identification of transcripts encoding putative transcription factors in cambium and differentiating xylem in *C. japonica *** **against *Arabidopsis thaliana***

**TF family**	**Description**	**vs Arabidopsis**	
		**ESTs**	%^*^
WRKY	WRKY DNA-binding domain	218	7.07
MYB	Myb-like DNA-binding domain	167	5.41
bHLH	Helix-loop-helix DNA-binding domain	162	5.25
SNF2	ATP binding/DNA binding/helicase/nucleic acid binding	150	4.86
PHD	Cys4--His--Cys3 zinc finger	148	4.80
Orphans	antiporter/multidrug efflux pump/transporter	135	4.38
C3H	Zinc finger, C-x8-C-x5-C-x3-H type	134	4.34
HB	Homeobox domain	131	4.25
AP2-EREBP	AP2 domain	130	4.21
C2H2	Zinc finger, C2H2 type	117	3.79
NAC	No apical meristem (NAM) domain	104	3.37
MYB-related	N-terminal myb-domain	83	2.69
SET	SET domain	76	2.46
bZIP	Basic leucine zipper (bZIP) motif	72	2.33
CCAAT	NUCLEAR FACTOR Y, SUBUNIT A10	61	1.98
ARF	Auxin response factor	56	1.82
G2-like	PRENYLATED RAB ACCEPTOR 1.G2	51	1.65
HSF	Heat shock factor	49	1.59
MADS	DNA-binding and dimaerization domain	47	1.52
GRAS	GRAS protein	45	1.46
C2C2-Dof	Dof zinc finger	45	1.46
FHA	Forkhead domain	45	1.46
ABI3VP1	ABI3/VP1 protein	43	1.39
Trihelix	Trihelix DNA-binding domain	42	1.36
GNAT	GCN5-related N-acetyltransferase (GNAT) family protein	39	1.26
Jumonji	nucleic acid binding/zinc ion binding	39	1.26
mTERF	mitochondrial transcription termination factor family protein	38	1.23
TRAF	TRAF homology domain-containing protein	36	1.17
C2C2-GATA	GATA zinc finger	32	1.04
LOB	LATERAL ORGAN BOUNDARIES	31	1.00
AUX/IAA	AUX/IAA family	25	0.81
DDT	DDT domain-containing protein	25	0.81
FAR1	N-terminal microtubule binding motor domain	25	0.81
ARID	AT-rich interaction domain	23	0.75
RWP-RK	RWP-RK domain-containing protein	23	0.75
SBP	SBP domain	23	0.75
CSD	superoxide dismutase	21	0.68
ARR-B	Arabidopsis response regulator B	20	0.65
DBP	protein phosphatase 2C	19	0.62
TAZ	TAZ zinc finger	18	0.58
zf-HD	zf-HD class homeobox domain	18	0.58
BES1	BRI1-EMS supressor	16	0.52
E2F-DP	DNA binding/protein heterodimerization	16	0.52
SWI/SNF-BAF60b	SWIB complex BAF60b domain-containing protein	16	0.52
TUB	structural constituent of cytoskeleton	16	0.52
C2C2-CO-like	CCT motif	15	0.49
CPP	copalyl pyrophosphate (CPP) of gibberellin biosynthesis	15	0.49
GRF	Growth regulation factor1	15	0.49
SWI/SNF-SWI3	chromatin binding/protein binding	14	0.45
GeBP	DNA-binding storekeeper protein-related	13	0.42
Pseudo ARR-B	Pseudo Arabidopsis response regulator B	13	0.42
TCP	ATP binding/protein binding	13	0.42
Tify	JASMONATE-ZIM-DOMAIN PROTEIN 6	13	0.42
BSD	BSD domain-containing protein	12	0.39
HMG	HMG (high mobility group) domain	12	0.39
LUG	LEUNIG gene	12	0.39
CAMTA	Calmodulin-binding transcription activators	11	0.36
PLATZ	Plant AT-rich sequnce and zinc-binding protein1	11	0.36
Alfin-like	Cys4 zinc finger and His/Cys3	10	0.32
EIL	Ethylene insensitivel (EIN3)	10	0.32
SRS	Domain unknown function	9	0.29
IWS1	molecular_function unknown	7	0.23
OFP	predicted nuclear localization signal	7	0.23
Sigma70-like	DNA binding/DNA-directed RNA polymerase	6	0.19
BBR/BPC	DNA binding	5	0.16
PBF-2-like	peptidase/threonine-type endopeptidase	5	0.16
VOZ	VOZ domain	4	0.13
LIM	LIM domain	3	0.10
RB	Retinoblastoma-associated protein B domain	3	0.10
Rcd1-like	RADICAL-INDUCED CELL DEATH1	3	0.10
SAP	STERILE APETALA domain	3	0.10
Coactivator p15	transcriptional coactivator p15 (PC4) family protein	2	0.06
C2C2-YABBY	YABBY transcription activator	2	0.06
S1Fa-like	DNA binding protein S1FA	2	0.06
HRT	nucleotide binding	1	0.03
LFY	Floricaula/Leafy protein	1	0.03
MBF1	Multiprotein bridging factor 1	1	0.03
MED6	RNA polymerase transcriptional regulation mediator-related	1	0.03
SOH1	SOH1 domain	1	0.03

^*^% is based on the 3085 Japanese cedar unique sequences with homologs in the PlnTFDB.

### Comprehensive gene expression changes during xylem formation

**Figure 5:**
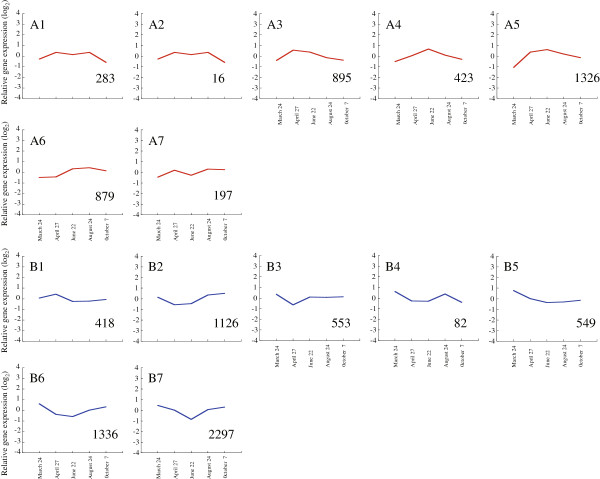
**Co-regulation patterns of differentially accumulated transcripts in xylem formation.** A total of 10,380 transcripts differentially accumulated in xylem formation were clustered into 14 groups using the Pearson correlation on the Subio platform. The graphs show the average expression profile of each cluster; changes are on a log_2_ scale. The gene expression pattern is shown as **A)** upregulation and **B)** downregulation during xylem formation. The description and expression profile of the individual targets are summarized in Additional file 2.

In Japanese cedar, physiological and anatomical alterations during cambial activity have been well investigated by anatomical observations [31-35], but little is known about the molecular changes that occur. In order to investigate expression of genes in Japanese cedar cambial tissue during xylem formation more comprehensively, we profiled transcripts at five time points during the growing season using microarray analysis. As a result, we identified 10,380 targets that were differentially regulated during xylem formation (*p* < 0.05, *q* < 0.2). The differentially expressed genes clustered into 14 different patterns based on their kinetics of gene expression (Figure 5, Additional file 2). These 14 patterns were divided into two expression patterns, associated with upregulation and downregulation, during xylem formation. Overall, we identified 4,019 targets that showed differential expression during the spring reactivation and the peak activity of xylem formation and 6,361 targets that showed differential expression during decreasing cell division and cessation of growth. Cluster A5 was the most abundantly transcribed during spring reactivation and the peak activity of xylem formation. Similarly, the group consisting of abundant clusters B2, 6, and 7 was notably observed during decreasing cell division/cessation of growth. The sequences of all targets in each cluster were also annotated against the COG database (Table 5) using the same settings as the cDNA library. Xylogenesis genes with well-known functions, such as in “Carbohydrate transport and metabolism (G),” “Cell wall/membrane/envelope biogenesis (M),” and “Cytoskeleton (Z),” were abundant categories in cluster A5, which indicated upregulation of these genes during this period. In the major group observed during decreasing cell division/cessation of growth, expressed genes related to tolerance of various conditions and to adjustment of cellular processes, such as “RNA processing and modification (A),” “Signal transduction mechanisms (T),” and “Defense mechanisms (V),” were abundant.

**Table 5:** Number of differentially expressed genes according to their cluster and functional COG classification

		**A1**	**A2**	**A3**	**A4**	**A5**	**A6**	**A7**	**B1**	**B2**	**B3**	**B4**	**B5**	**B6**	**B7**
[A]	RNA processing and modification	3	0	6	3	11	21	0	3	23	9	4	15	60	123
[B]	Chromatin structure and dynamics	1	0	7	0	13	2	4	8	10	1	1	5	18	78
[C]	Energy production and conversion	20	0	25	26	37	29	0	8	31	18	0	6	32	10
[D]	Cell cycle control, cell division, chromosome partitioning	1	0	8	4	23	5	0	3	12	3	0	2	20	21
[E]	Amino acid transport and metabolism	7	2	32	11	39	28	4	10	23	16	1	7	8	53
[F]	Nucleotide transport and metabolism	0	0	2	0	12	11	0	1	8	2	2	4	6	15
[G]	Carbohydrate transport and metabolism	9	0	65	26	113	41	9	29	32	23	2	19	69	47
[H]	Coenzyme transport and metabolism	11	0	2	7	19	7	1	1	8	2	0	1	3	5
[I]	Lipid transport and metabolism	6	0	34	10	53	33	9	13	37	18	1	18	17	40
[J]	Translation, ribosomal structure and biogenesis	7	0	28	1	11	8	0	35	9	5	0	4	13	42
[K]	Transcription	15	2	19	7	30	23	3	12	59	24	2	20	91	98
[L]	Replication, recombination and repair	0	0	8	5	8	4	2	7	16	4	1	5	18	35
[M]	Cell wall/membrane/envelope biogenesis	2	1	29	9	65	17	5	11	19	3	0	3	14	38
[N]	Cell motility	0	0	0	2	1	0	0	0	1	0	0	0	1	2
[O]	Posttranslational modification, protein turnover, chaperones	18	0	30	37	51	84	29	21	44	33	10	20	62	110
[P]	Inorganic ion transport and metabolism	9	0	24	8	11	15	5	7	8	8	1	9	12	48
[Q]	Secondary metabolites biosynthesis, transport and catabolism	8	2	17	11	50	31	9	18	68	36	1	7	36	48
[R]	General function prediction only	16	0	104	52	133	121	26	45	159	60	7	50	132	263
[S]	Function unknown	18	0	61	23	87	39	5	22	37	50	4	33	68	69
[T]	Signal transduction mechanisms	23	4	97	22	126	40	32	51	77	40	10	88	144	435
[U]	Intracellular trafficking, secretion, and vesicular transport	5	0	48	26	45	17	3	20	12	6	0	8	25	28
[V]	Defense mechanisms	2	0	8	8	35	19	7	19	20	15	3	55	64	227
[W]	Extracellular structures	1	0	2	1	22	2	1	0	0	0	0	0	1	3
[X]	Unassigned	21	2	70	25	123	67	24	19	87	36	5	43	98	146
[Y]	Nuclear structure	1	0	3	0	1	0	1	5	1	0	0	3	0	9
[Z]	Cytoskeleton	4	0	32	19	41	9	0	14	5	1	0	9	15	8

### Cell-cycle related genes

**Figure 6:**
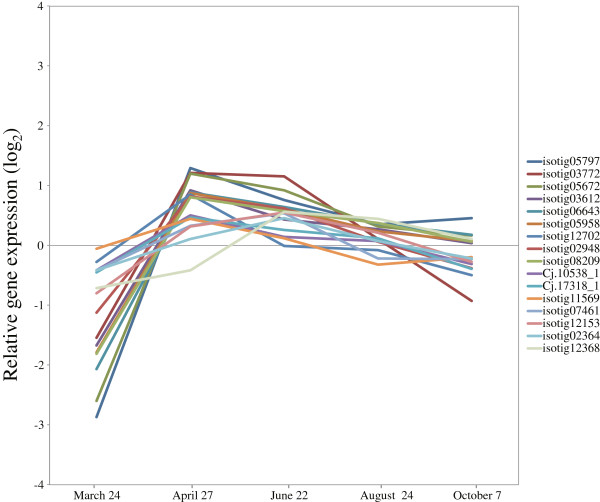
**Expression of cell-cycle genes in cambial region during xylem formation.** The individual targets are summarized in Additional file 3. All expression data are presented on a log_2_ scale.

Druart et al [36] reported the expression of 68 homologs of aspen trees based on 80 core cell-cycle genes that were investigated in *Arabidopsis*[37]. The expression patterns did not correspond to the increasing number of dividing cambial cells during the early phase of cambial cell-cycle activation, leading to the hypothesis of posttranscriptional control of expression after cessation of growth [36]. Similarly, we investigated the expression of *Arabidopsis* core cell-cycle gene homologs in Japanese cedar. We observed upregulation of 16 of 25 genes from March to April, which suggested that the activation of cell division and induction of cell-cycle genes are correlated in early activity during xylem formation (Figure 6, Additional file 3). Our findings agreed well with data from *Arabidopsis*[37] and poplar, for which the expression of *CDKB* and *CYCB* is regulated seasonally following a rise in temperature [38]. Our data suggested that cambial reactivation occurred in Japanese cedar between 24 March and 27 April based on anatomical observation. To prove the hypothesis of posttranscriptional control of expression after cessation of growth, we therefore harvested samples during this period.

### Xylogenesis genes related to phenylpropanoid metabolism

During the development of xylem tissue, primary cell wall biosynthesis, secondary wall deposition, and lignification are important fundamental processes, because of the need for maintaining biological mechanisms conferring adaptability to various environments, compressive strength and defense against pathogens. These processes are also important determinants of wood properties.

**Figure 7:**
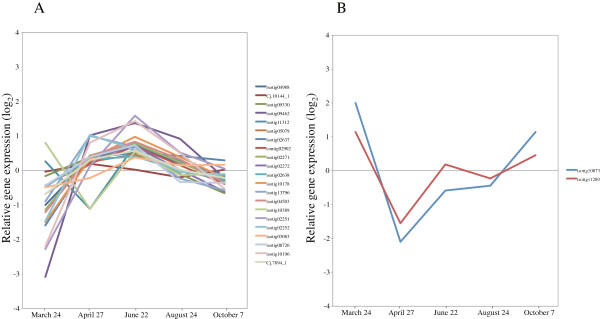
**Expression of phenylpropanoid metabolism-related genes in cambial region during xylem formation. A)** Upregulated genes involved in lignin biosynthesis. **B)***PAL4* (isotig 10873) and *4CL3* (isotig 11289). The individual targets are summarized in Additional file 3. All expression data are presented on a log_2_ scale.

The identification and expression profiling of gene family members that are responsible for developmental lignification have been reported for *P. trichocarpa*, *Picea abies*, and *Pinus teada*[39-41]. In our study, the expression of the most of these gene family members (*Phenylalanine ammonia-lyase* (*PAL*); *4-coumarate: CoA ligase* (*4CL*); *Cinnamate-4-hydroxylase* (*C4H*); *Hydroxycinnamoyl: CoA shikimate/quinate hydroxycinnamoyl transferase* (*HCT*); *p-coumarate-3-hydroxylase* (*C3H*); *Caffeoyl-CoA O-methyltransferase; Cinnamyl alcohol dehydrogenase* (*CCoAOMT*); *Cinnamoyl-CoA reductase* (*CCR*); and *Cinnamyl alcohol dehydrogenase* (*CAD*)) was induced from March to April, and then expression gradually decreased from the peak activity of xylem formation through August (Figure 7A, Additional file 3). The expression of these genes corresponded to our anatomical observation that the number of cambial cells rapidly increased from March to June (Figure 1A,B). These observations indicate that these genes are the main transcripts in developing xylem of Japanese cedar.

Interestingly, enzymes in the early part of the monolignol pathway, acting between *PAL* and *4CL*, are also involved in the biosynthesis of other phenylpropanoids, like flavonoids, coumarins, and stilbene [39]. Lignans, which are monolignol-derived dimers and oligomers involved in such processes as defense reactions, are synthesized through the same pathway [39]. The expression of *PAL4* (isotig 10873) and *4CL3* (isotig 11289) was upregulated during dormancy and following cessation of growth (Figure 7B, Additional file 3), which indicates that these enzymes could play roles in defense, such as responses to infection, wounding, drought stress and temperature change.

**Figure 8:**
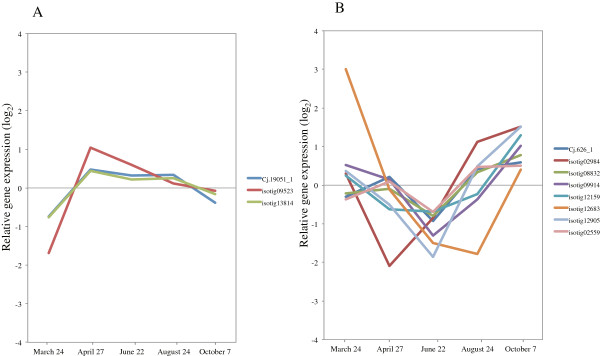
**Expression of peroxidase superfamily in cambial region during xylem formation. A)** Genes upregulated in activity period. **B)** Genes upregulated during dormancy and cessation of growth. The individual targets are summarized in Additional file 3. All expression data are presented on a log_2_ scale.

Lignins result from the oxidative polymerization of *p*-hydroxycinnamyl alcohols, which can be mediated by both laccase and peroxidase [42]. For the 19 peroxidase superfamily proteins that we examined, the levels of 3 transcripts (isotigs 09523, 13814, and Cj.19051_1) increased during peak xylem formation (Figure 8A, Additional file 3), which corresponded to anatomical observations. The peroxidases that were induced during this period are the strongest candidates for involvement in lignin polymerization. The expression of 7 peroxidase superfamily proteins was upregulated during dormancy and correlated with cessation of growth (Figure 8B, Additional file 3). In *P. abies* and *Pinus sylvestris*, high peroxidase activities have also been measured outside the growth period during late autumn, winter, and early spring [43]. Some peroxidase genes generally respond to external stimuli such as wounding, UV-irradiation, bending stress and pathogen infection [39,44]. These previous reports suggest functions of these genes during the inactive period for the cambium.

**Figure 9:**
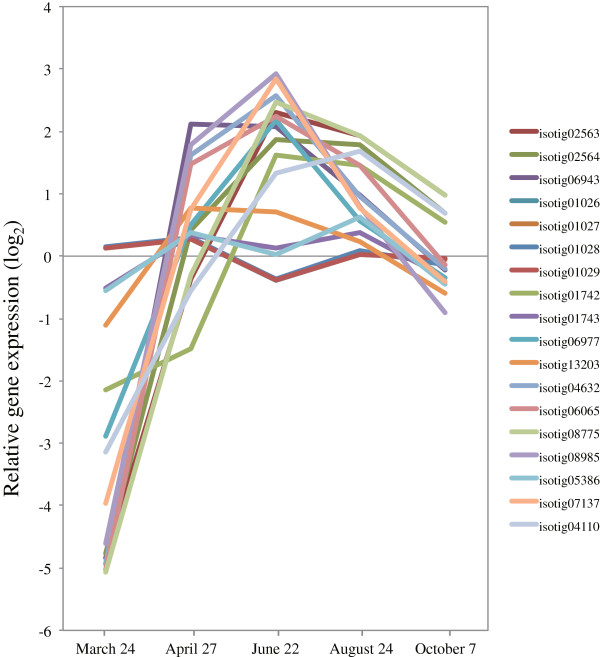
**Expression of laccase genes in cambial region during xylem formation.** The individual targets are summarized in Additional file 3. All expression data are presented on a log_2_ scale.

Recently, it was reported that laccase genes *Lac4* and *Lac17* contribute to constitutive lignification in an *A. thaliana* mutant [45]. Expression of laccase was 4–5 times higher than peroxidase in developing xylem and young vertical xylem in *P. abies*[39]. The induction of *LAC17* (isotigs 04632, 06065, 08775, and 08985) and *LAC4* (isotig 04110) increased rapidly at the peak activity of xylem formation in comparison to other lignification-related genes (Figure 9, Additional file 3). These most highly expressed laccases are also candidates for involvement in lignin polymerization.

### Xylogenesis genes related to carbohydrate, cellulose, and hemicellulose metabolism

**Figure 10:**
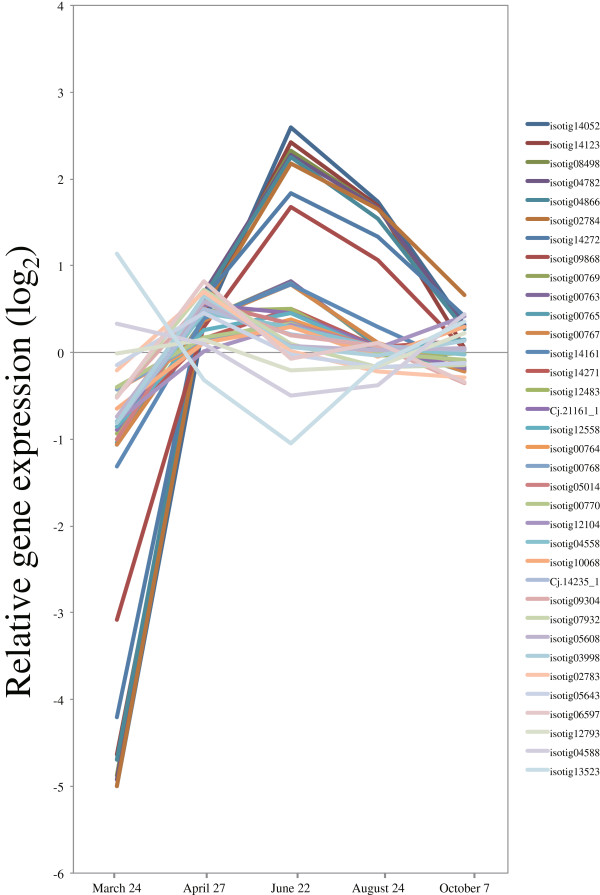
**Expression of cellulose synthase and cellulose synthase-like genes in cambial region during xylem formation.** The individual targets are summarized in Additional file 3. All expression data are presented on a log_2_ scale.

**Figure 11:**
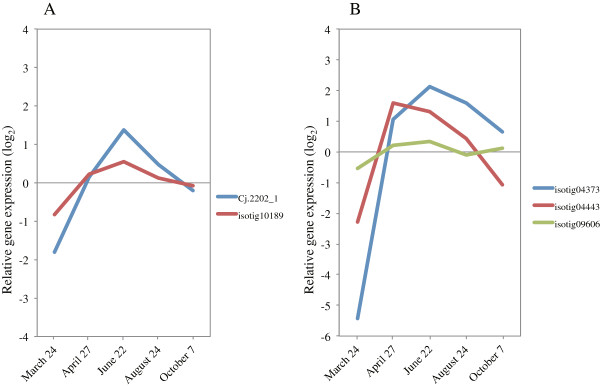
**Expression of *KORRIGAN1 *and *COBRA *in cambial region during xylem formation. A)***KORRIGAN1* (*KOR1*). **B)***COBRA* (*COB*). The individual targets are summarized in Additional file 3. All expression data are presented on a log_2_ scale.

Cellulose is generally the main component of the plant cell wall, and is synthesized at the plasma membrane by a large multimeric cellulose synthase (*CesA*) complex [46]. The patterns of expression for almost all expressed *Cellulose synthase* and *Cellulose synthase-like* (*Csl*) genes that were upregulated during peak activity of xylem formation are shown (Figure 10 and Additional file 3). Of these genes, *CesA1* (isotig 04782), *Ces4/IRX5* (isotigs 14272, 08498), *CesA6* (isotig 14123), *CesA7/IRX3* (isotigs 04866, 09868), and *CesA8/IRX1* (isotigs 02784, 14052) were rapidly induced from March to April, and then their expression gradually decreased from the period of peak xylem formation until cessation of growth (Figure 10, Additional file 3), which corresponded with anatomical observations. Interestingly, *Ces4/IRX5*, *CesA7/IRX3* and *CesA8/IRX1* are required for cell-wall biosynthesis in vascular tissue of *Arabidopsis* and rice [47,48]. Similarly, orthologs identical to these three genes are involved in secondary cell-wall biosynthesis in developing xylem of wood species such as *Populus* and *Pinus*[14,49-53]. These findings suggest that the functional roles of these orthologs are conserved in cell-wall synthesis of vascular tissue in herbaceous and woody dicotyldeons, monocotyledons and gymnosperms [14]. Additionally, a membrane-bound endoglucanase, *KORRIGAN1* (*KOR1*), and a glycosylphosphatidylinositol-anchored protein, *COBRA* (*COB*), have been implicated in cellulose biosynthesis in *Arabidopsis*[54,55]. Both the sequence of the orthologs and their functional roles are reportedly conserved in *Populus* and *Picea*[56,57]. The Japanese cedar homologs of *KOR1* and *COB* were upregulated during peak xylem formation, suggesting a conserved functional role (Figure 11A,B, Additional file 3).

**Figure 12:**
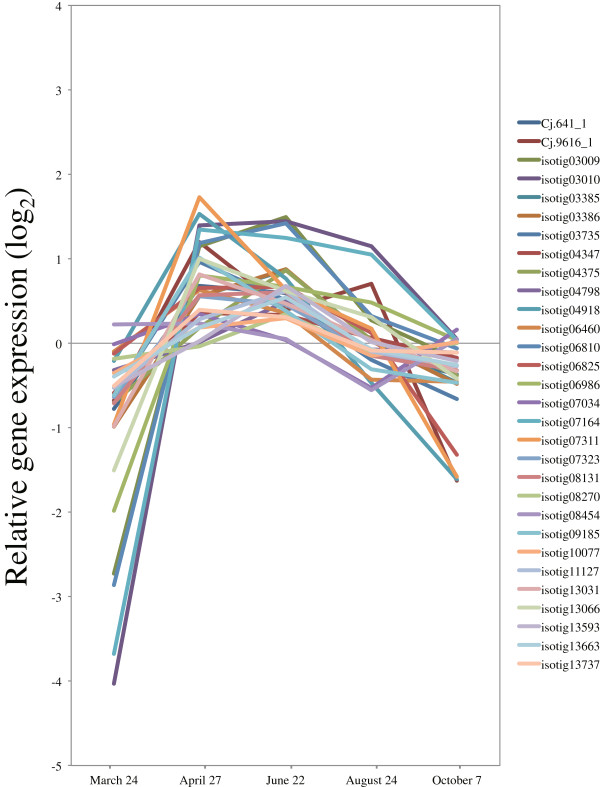
**Expression of hemicellulose-related gene family in cambial region during xylem formation.** The individual targets are summarized in Additional file 3. All expression data are presented on a log_2_ scale.

**Figure 13:**
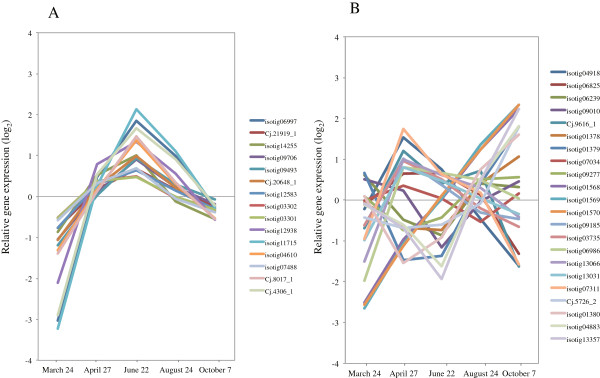
**Expression of glycosyltransferase and xyloglucan endotransglycosylase in cambial region during xylem formation. A)** Glycosyltransferase. **B)** Xyloglucan endotransglycosylase. The individual targets are summarized in Additional file 3. All expression data are presented on a log_2_ scale.

Hemicelluloses, including glucomannans and xyloglucans, are major components of the plant secondary cell wall. Most genes related to the glucomannan/galactomannan pathway (such as *GDP-D-mannose 4,6-dehydratase1, mannose-1-phosphate guanylyltransferase, phosphomannomutase, mannosyltransferase family protein, galactosyltransferase family protein*) and to synthesis of xylan (such as *UDP-glucuronic acid decarboxylase: UDP-xylose synthase, β-(D)-xylosidase*) and xyloglucan (such as *xyloglucan endotransglycosylase, xyloglucan endotransglycosylase/hydrolase*) were induced during xylem formation, which suggests that the encoded proteins play an active role in secondary wall formation (Figure 12, Additional file 3). Recently, a number of genes encoding putative glycosyltransferases required for xylan synthesis or deposition have been identified in *Arabidopsis* using knockout mutants [58-63]. The expression of most of these genes (*IRX7/FRA8*, *IRX9*, *IRX10-like*, *IRX14*, and *IRX15*) increased during xylem formation, indicating conserved functional roles of these orthologs (Figure 13A, Additional file 3). Xyloglucan is incorporated and modified in the cell-wall network by xyloglucan endotransglycosylases and hydrolases (XTHs, also known as XET/hydrolases and XEHs) [64,65]. We observed 10 genes involved in cell-wall biosynthesis to be upregulated at peak xylem formation; however, the expression of 12 genes was downregulated (Figure 13B, Additional file 3). Some *XTH* genes are induced in dormant cambium and cold-stressed organs [65]. In Japanese cedar, these 12 genes may be candidates for this functional role.

**Figure 14:**
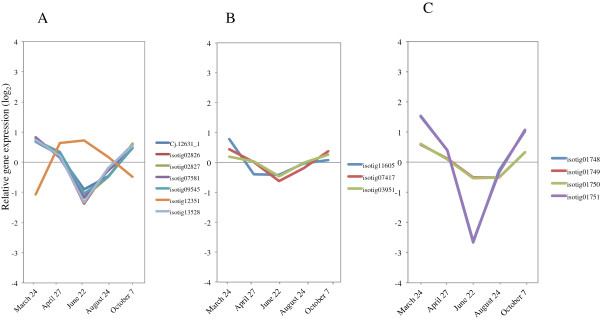
**Expression of sucrose synthase, invertase, and sucrose phosphate synthase in cambial region during xylem formation. A)** Sucrose synthase. **B)** Invertase. **C)** Sucrose phosphate synthase. The individual targets are summarized in Additional file 3. All expression data are presented on a log_2_ scale.

Sucrose synthase (Sus) catalyzes formation of UDP-glucose, the immediate substrate for cellulose biosynthesis. Members of the *Sus* gene family in many plant species are divergent in function and differentially expressed during plant development [66]. In *Pinus* and *Populus*, some *Sus* genes showed an expression pattern identical to that of *Ces* genes in developing xylem [5,14]. Similarly, the expression of *Sus* (isotig 12351) was upregulated from April to June, which suggested it as a robust candidate gene for involvement in xylem formation (Figure 14A, Additional file 3). Interestingly, all other *Sus* genes were rapidly downregulated from March to June, and then expression gradually increased through October. *Sus* gene activity is considered to be associated with environmental stresses, such as cold, drought and O_2_ deficiency [36,67]. We observed upregulation of these genes in March and October, months showing markedly low temperature, which suggested synthesis of cryoprotectants and responses to cold stress (Figure 14A, Additional file 3). Additionally, because reactivation of the cambium in the spring occurs before any significant photosynthesis activity, the induction of the *Sus* gene and various invertases indicates that sucrose catabolism generates hexose that can be metabolized via glycolysis during this period [36]. The reaction catalyzed by sucrose phosphate synthase (SPS) plays an important regulatory role in controlling *Sus* genes in plants [68]. In hybrid poplar, an *AtSPS* transgenic hybrid has altered phenology, such as timing of leaf senescence and bud break, compared to wild type [69]. Some invertase and *SPS* genes also showed an expression pattern identical to that of the *Sus* genes (Figure 14B,C, Additional file 3). In Japanese cedar, these expression profiles could indicate that these genes are involved in providing an alternative source of energy and carbon skeletons in the early period of cambial reactivation in spring.

### Transcription factors

**Figure 15:**
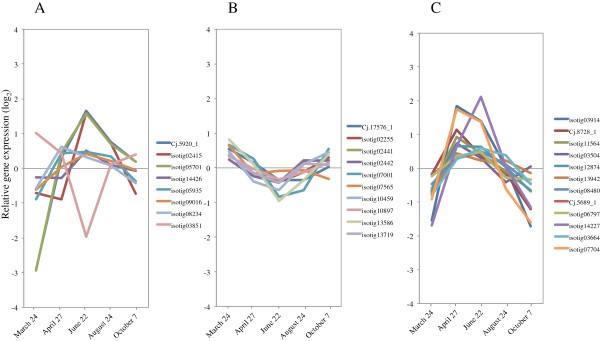
**Expression of cell wall-related transcription factors in cambial region during xylem formation. A)** MYB. **B)** NAC. **C)** Other cell wall-related transcription factors (LIM, HB, b-ZIP, WRKY). The individual targets are summarized in Additional file 3. All expression data are presented on a log_2_ scale.

Several transcription factor family members, such as NAC, MYB, zinc finger proteins, and proteins with a Lim domain or a homeodomain are thought to help regulate secondary cell-wall biosynthesis [70-76]. In particular, some transcription factors of the NAC and MYB subfamilies are master switches in the transcriptional network for secondary cell-wall biosynthesis [75]. In *Arabidopsis*, some MYB genes (*AtMYB20*, *43*, *46*, *52*, *63*, *83*, *85*, *99*, *103*, and *118*) are significantly upregulated in expression just when xylem vessel elements actively form [77]. In conifers, *Picea* (*PgMYB2*, *4*, *8*) and *Pinus* (*PtMYB1*, *4*) MYB genes are also involved as transcriptional regulators in lignin metabolism and/or wood formation in stem and root [78-80]. Most of these *MYB* genes are clustered in a phylogenetic tree of MYBs from spruce, *Pinus*, *Arabidopsis*, and the nearest sequences from other species [78]. We found that 34 MYB genes were upregulated during the peak activity of xylem formation (Figure 15A, Additional file 3). *AtMyb20* (isotig 05701) and *AtMyb43* (Cj.5920_1) were expressed preferentially; however, *AtMyb103* (isotig 03851) was downregulated during this period. These findings suggest that the functional roles of *MYB20* and *MYB43* orthologs are conserved in cell-wall synthesis of vascular tissue in Japanese cedar and other species. Recent molecular and genetic studies have revealed that a subgroup of *Arabidopsis* NAC domain transcription factors (*SND1*, *NST1*, *VND6*, *7*) are master switches regulating a cascade of downstream transcription factors, leading to activation of secondary wall biosynthesis [81-86]. Although among these NAC domains, only a *VND6* homolog (Cj17576_1) was included on our array; its expression was moderately decreased, as with 9 NAC family homologs (*anac2*, *8*, *28*, and *45*) during peak xylem formation (Figure 15B, Additional file 3). The homologs of other cell-wall biosynthesis-related transcription factors (LIM, HB, b-ZIP, WRKY) were induced during this time (Figure 15C, Additional file 3), implying that these genes could be important in regulating downstream genes.

### Hormonal regulation of the activity-dormancy cycle

**Figure 16:**
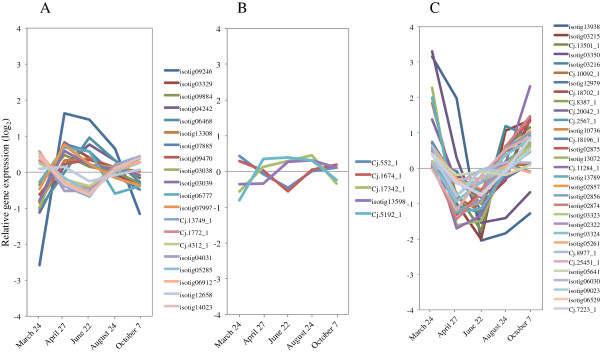
**Expression of hormonal regulation-related genes in cambial region during xylem formation. A)** Auxin signaling and transport component. **B)** GA biosynthesis-related and signaling genes. **C)** ABA biosynthesis-related and signaling genes. The individual targets are summarized in Additional file 3. All expression data are presented on a log_2_ scale.

Auxin has been implicated as a key signal regulating cambial cell proliferation and cambial meristem identity [12]. In the cambial region, the amount of IAA varies seasonally, and rapid induction in cambial activity occurs in spring to early summer [87-89]. The IAA distribution shows a radial gradient and is most concentrated in the cambial region [90,91]. The positive correlation observed between the regions with high IAA levels and the number of cells in the same region suggests that the gradient in endogenous IAA level controls the number of cambial cells [92]. The expression of some auxin signaling and transport component genes (*Aux1*, *IAA16*, *27*, *Auxin efflux carrier* (*PIN1, 2*), *Auxin response factor* (*ARF1, 2, 4*), and *SAUR-like auxin-responsive protein*) was upregulated in April, and then gradually decreased through October (Figure 16A, Additional file 3). The concentration of IAA in *P. sylvestris* is high at the start of cambial reactivation, declines when the number of differentiating tracheids begins to increase, and then rises as the number of cells decreases [88]. In Japanese cedar, our results indicate that these genes are regulated early in xylem formation. Auxin signaling is mediated through the ubiquitin-proteasome pathway, in which AUX/IAA proteins are degraded through SCF^TIR1^ complexes, composed of cullin, SKP1, F-box protein, and RBX1 protein [93,94]. We observed inverse expression of these genes relative to the expression of some auxin signaling and transport component genes, suggesting that auxin signaling and transport component genes are repressed during cessation of growth.

Gibberellins (GAs) act synergistically with auxin in stimulating cambial growth [95]. The analysis of transgenic aspen indicated that GAs are required both in xylogenesis, which is likely mediated by GA signaling in the cambium, and in fiber elongation in the developing xylem [96]. In angiosperm trees, application of GA results in the formation of wood fibers with enhanced thickness of the inner layers of cell walls [97,98]. We found that a homolog of GA3-oxidase (GA3ox, Cj.17342_1), implicated in the last step of GA biosynthesis, and the receptor gene *GID1* (Cj.5192_1, isotig13598) were moderately upregulated at peak xylem formation (Figure 16B, Additional file 3). The genes encoding GA biosynthetic enzymes GA20-oxidase (GA20ox) and GA3ox are particularly important for control of bioactive GA levels [99]. GA signaling operates as a derepressible system that is moderated by DELLA-domain proteins, which are transcriptional regulators that repress GA responses [99]. Like DELLA-domain proteins, the homologs of *RGA* (Cj 552_1, 1674_1) were expressed inversely to these genes (Figure 16B, Additional file 3). These findings suggest that genes involved in GA signaling have an important role in xylem formation.

Abscisic acid (ABA) content increases during abiotic stress, and especially protects plant water status. In poplar cambium, ABA levels are increased by short days and by short days with low temperature in late autumn and during cambial reactivation in early spring [36]. Genes related to ABA biosynthesis and signaling, such as *ABA4*, *NCED*, *CYP707A*, *PP2C* (*HAI1*, *2*), *SnRK2.6* (*OST1*), *ABRE* (*ABI1*, *5*, *ABF1*), and *PYL* (*1*,*4*,*10*), were upregulated in March and October (Figure 16C, Additional file 3). Most of these genes were rapidly downregulated from March to April, suggesting that their downregulation is coincident with release from cold hardiness and the improvement in water deficit on cambial reactivation. The *Arabidopsis CYP707A* gene family (*CYP707A1*, *2*), involved in ABA catabolism, controls seed dormancy [100]. Therefore, our observations suggest that ABA is degraded during cambial reactivation in Japanese cedar. In the apex of hybrid aspen, some *9-cis-epoxycarotenoid* genes (such as *NCED*), which are involved in ABA biosynthesis, are induced after 5 weeks of short-day treatment, which also induces growth cessation [101]. As seen in our data (Figure 16C, Additional file 3), these genes (Cj13501_1, 2567_1, 8387_1) were upregulated in accordance with changes in day length. Other ABA biosynthesis-related and signaling genes were also upregulated from August to October, indicating they may be induced in response to several abiotic stresses (such as cold and drought) that also lead to cessation of growth (Figure 16C, Additional file 3).

### Development of cold hardiness in activity-dormancy cycle

**Figure 17:**
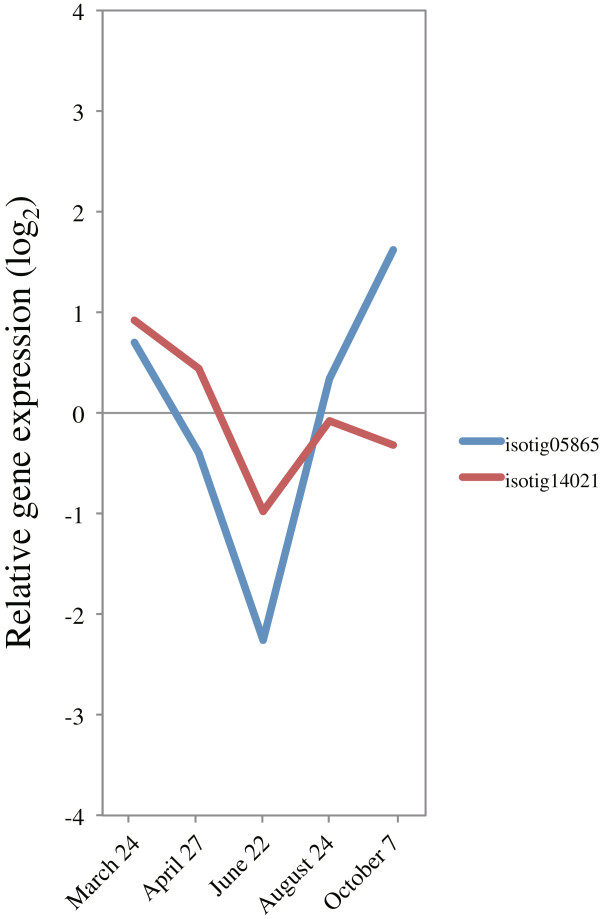
**Expression of *ICE1 *in cambial region during xylem formation.** The individual targets are summarized in Additional file 3. All expression data are presented on a log_2_ scale.

**Figure 18:**
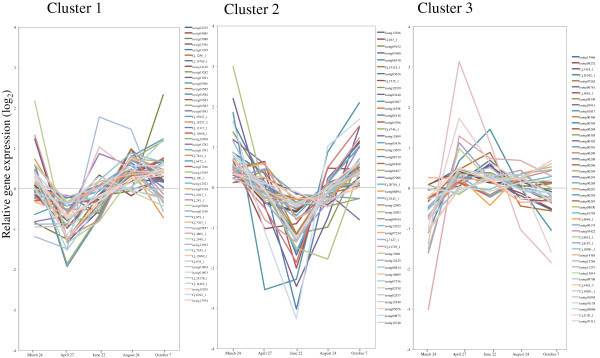
**Expression of low temperature-induced genes in cambial region during xylem formation.** Low temperature-induced and cold hardiness-related genes were clustered using the Pearson correlation on the Subio platform into three main patterns of expression during the autumn transition and early spring (clusters 1, 2) and during spring reactivation (cluster 3). The individual targets are summarized in Additional file 3. All expression data are presented on a log_2_ scale.

On 24 March, before cambial reactivation, cold hardiness was maintained in cambial cells (Figure 1A,B). On the other hand, the number of expanding cells and cells depositing secondary walls, as well as temperature and day length, rapidly decreased from 24 August to 7 October (Figure 1A,B, Additional file 4: Figure S2), suggesting acquisition of cold hardiness on growth cessation. The transcriptional regulators and modulating genes involved in the acquisition of cold hardiness of Japanese cedar have not been identified. In *Arabidopsis*, the *ICE1* (*inducer of CBF expression*) and CBF (*C-repeat binding factor*) family transcription factors are respectively upstream and downstream regulators of the cold-responsive transcriptome and freezing tolerance [102,103]. The role of the CBF family as transcriptional activators in cold acclimation of *Arabidopsis* has been maintained in *Populus*; in particular, *CFB1* and *CFB3* show significant induction in *Populus* stems [104]. The only *ICE1* homologs presented on our array were induced in conditions consistent with maintaining and acquiring cold hardiness (isotigs 05865, 14021) (Figure 17, Additional file 3), so these genes are candidates for this functional role. Druart *et al*. [36] listed and clustered the expression pattern of genes involved in cold hardiness from three data sets: 1. poplar genes induced by low temperature and *atCBF* overexpression; 2. a poplar homolog of an *Arabidopsis* gene induced by low temperature; and 3. poplar homologs of genes involved in the development of cold hardiness in three other tree species [36]. We clustered the expression patterns of these genes based on their timing of induction. Clustering of the 138 homologs of these genes yielded three main groups, two associated with autumn transition and early spring (clusters 1, 2), and another with spring reactivation (cluster 3) (Figure 18, Additional file 3). Their expression rapidly decreased from March to April, which suggested repression and release of cold hardiness in cluster 1. In cluster 2, expression (for example, of *Cold regulated gene* and *dehydrin family protein*) was up- or downregulated in accordance with changes in day length. In acquisition of cold hardiness, these genes were moderately upregulated prior to reduction in temperature (Additional file 4: Figure S2). This finding implies that a signal other than low temperature (such as short days) must trigger the induction of these genes in autumn under natural conditions [36,105]. In cluster 3, some homologs were superinduced from March to April, which corresponded to previous findings [36]. The exact role of this superinduction is unclear; however, it might reflect a need to protect the very sensitive dividing cambial cells from sudden drops in temperature during early spring [36].

**Figure 19:**
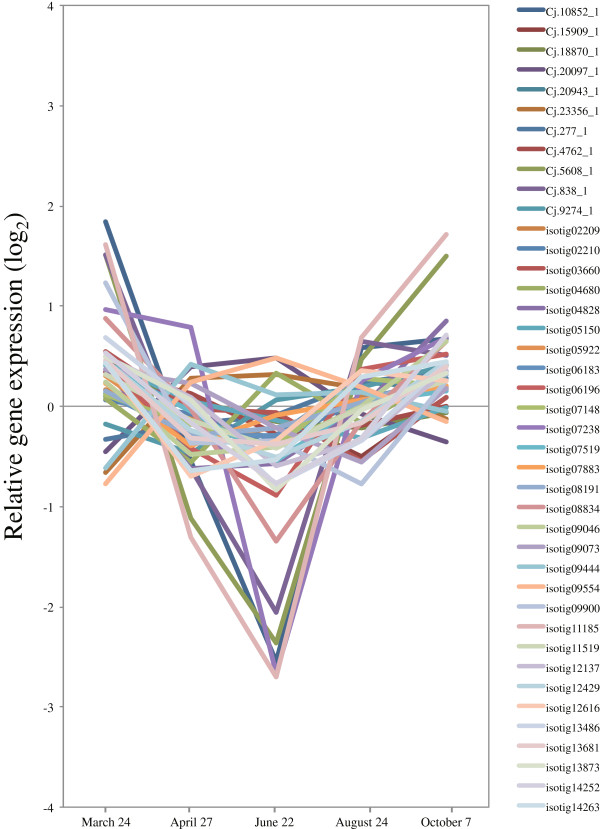
**Expression of starch-breakdown related genes in cambial region during xylem formation.** The individual targets are summarized in Additional file 3. All expression data are presented on a log_2_ scale.

Because of limited photosynthesis in autumn, plants must derive the energy and carbon required for the acquisition of cold hardiness from some other source [36]. Conversion of starch to sugar is a key metabolic process associated with the entry into dormancy, as starch-derived sugars serve several purposes, for example as cryoprotectants as well as a source of energy [12]. Transcriptional induction of a key enzyme of the starch breakdown pathway occurs in poplar cambium in autumn and during dormancy [12,36]. Our data showed that most homologs involved in the process, such as *β-amylase* and *phosphoglucan-water dikinase*, were induced in March and October (Figure 19, Additional file 3). These profiles suggested them as candidate genes for regulating the availability of an alternative energy and carbon source during limitations on photosynthetic activity.

### Cytoskeleton-related genes

**Figure 20:**
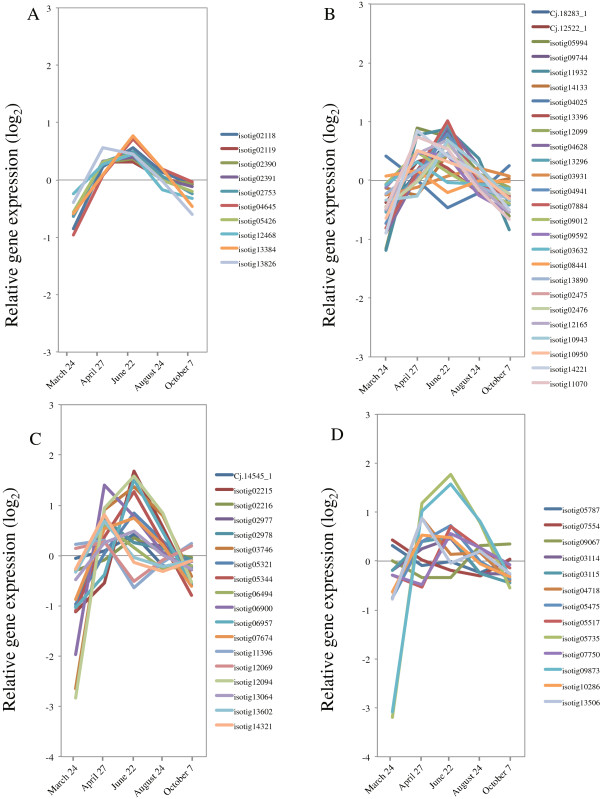
**Expression of cytoskeleton related genes in cambial region during xylem formation. A)** α/β-Tubulin. **B)** Actin, actin-related and actin interacting proteins. **C)** Kinesin gene family. **D)** Microtubule-associated protein gene family. The individual targets are summarized in Additional file 3. All expression data are presented on a log_2_ scale.

The cytoskeleton regulates cellular polarity, morphology, and movement through its involvement in cellular events such as vesicular traffic, organellar movement, abiotic and biotic stress sensing, signal transduction, and cell wall biosynthesis [106]. Plant cell morphogenesis relies on the organization and function of two polymer arrays separated by the plasma membrane: the cortical microtubule cytoskeleton and cellulose microfibrils in the cell wall [107]. In the large S2 layer of secondary fiber cell walls, the orientation of microfibril deposition, which is directed by cortical microtubules, is an important trait determining wood quality and wood stiffness or elasticity, and is referred to in trees as the microfibril angle [108]. The expression of genes for *α*- and *β-tubulins*, which comprise dynamic arrays of cortical microtubules, appear to play a role in determining these characteristics during xylem development in *Populus* and *Eucalyptus*[108,109]. Our data showed that most *tubulin* gene family members are highly expressed during peak xylem formation (Figure 20A, Additional file 3). Similarly, most other cytoskeleton-related genes such as *Actin* and genes encoding *actin-related* or *-interacting proteins* (*actin binding protein*, *actin related protein*, *actin depolymerization protein*, *villin*, *fibrin profiling*, *capping protein*), *microtubule-associated protein* (*MAP*), *microtubule-motor family protein*, *microtubule end binding protein*, and *kinesin* were also induced (Figure 20B,C,D, Additional file 3).

Actin forms microfilament structures by self-polymerization and interactions with numerous actin-binding proteins. In our data, the four homologs of *atACT7* (isotigs 05994, 09744, 11932, and 14133) were upregulated during peak xylem formation, along with actin-related/interacting protein and a gene encoding a kinesin family protein (Figure 20B,C, Additional file 3). The homologs clustered closely in a group with a *Pinus* homolog in a phylogenetic tree of *actin* from *Arabidopsis* and the nearest sequences from other species [110]. *atACT7* is preferentially expressed in younger, rapidly developing tissue, such as during germination and root growth in *Arabidopsis*[110,111]. These findings correspond with our findings in developing xylem.

The organization and dynamics of microtubules are regulated by *MAPs*[108]. Our study found a gene encoding a *MAP* (*MAP65-1*: isotig 05735, 09873) that was more strongly transcribed than other *MAP* genes (Figure 20D, Additional file 3). *AtMAP65-1* is able to promote tubulin polymerization, enhance microtubule nucleation, and decrease the critical concentration for tubulin polymerization [112]; this role agrees reasonably well with what would be expected from our expression pattern and anatomical observations.

### Validation of microarray expression of 12 selected genes by qRT-PCR

**Figure 21:**
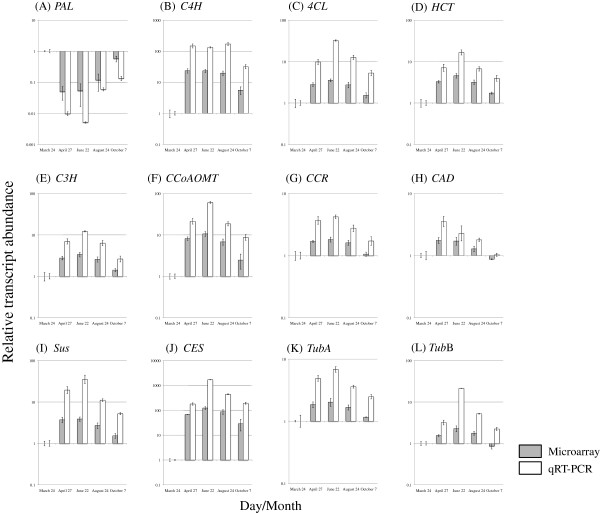
**Validation of microarray expression of 12 selected genes by qRT-PCR.** A total of 12 genes were selected in the validation using qRT-PCR: **(A)***Phenylalanine ammonia-lyase* (*PAL:* isotig 10873), **(B)***Cinnamate-4-hydroxylase* (*C4H*: isotig 09462), **(C)***4-Coumarate:CoA ligase* (*4CL*: isotig 04988), **(D)***Hydroxycinnamoyl-CoA shikimate/quinate hydroxycinnamoyl transferase* (*HCT*: isotig 10178), **(E)***p-Coumarate-3-hydroxylase* (*C3H*: isotig 02271), **(F)***Caffeoyl-CoA O-methyltransferase; Cinnamyl alcohol dehydrogenase* (*CCoAOMT*: isotig 10196), **(G)***Cinnamoyl-CoA reductase* (*CCR*: isotig 05079), **(H)***Cinnamyl alcohol dehydrogenase* (*CAD*: isotig 02638), **(I)***Sucrose synthase* (*Sus*: isotig 12351), **(J)***Cellulose synthase* (*Ces*: isotig 08498), **(K)** α-*Tubulin* (*Tub α:* isotig 02753), **(L)***β−Tubulin* (*Tub β:* isotig 13384). In qRT-PCR, *Ubiquitin* (UBQ) was used as a reference gene, and the data were calibrated relative to transcript levels in the 24 March sample. Error bars show standard deviation for three replicates.

Microarray expression data of 12 differentially transcribed genes selected in this study were validated by qRT-PCR using the same RNA samples used for the microarray experiments. Transcript accumulation measured by qRT-PCR was fairly consistent with the microarray results for all 12 validated genes (Figure 21), particularly in the ranking of magnitude of expression, indicating that the microarray experiments in this study were sufficiently reliable for the identification of genes that may influence xylem formation in the cambium of Japanese cedar.

## Conclusions

In this study, we obtained 55,051 unique sequences by sequencing a non-normalized cDNA library from the cambial meristem and derivative cells of Japanese cedar. A custom cDNA microarray was designed based on this library and EST data to investigate seasonal gene expression in Japanese cedar. This is the first comprehensive study of an extensive collection of EST sequences and expression studies related to xylem formation in Japanese cedar. Because Japanese cedar belongs to a different lineage than the *Pinaceae*, comparison of data could lead to significant findings on genome evolution in coniferous species. Our data may also be a useful resource for forward genetics and functional genetics studies in wood species.

## Methods

### Plant material

Tissue from the cambium region (including phloem and the differentiating xylem) was taken from four 15-year-old trees of *Cryptomeria japonica* plus-trees, clones Chousui8, Iiyama9, Nisihkawa10 and Tano1, in Hitachi, Ibaraki Prefecture for molecular analysis. The daily minimum and maximum temperatures were also recorded during the study (Additional file 4: Figure S2). The harvested tissues were immediately frozen in liquid nitrogen in the field, and then stored in the laboratory at −80°C for later RNA extraction. A square block (approximately 1 cm^2^) was collected for microscopy and fixed in FAA (formalin: acetic acid: 50% alcohol, 5:5:90) in the field. To evaluate how gene expression and morphological development in the cambial region changed over a single growing season, tissues from this region were collected from different trees at the same time (around 10 AM) on 15 different dates from 2010 to 2011: on 9 March, 9 April, 10 May, 1 June, 24 June, 16 July, 16 August, 19 September, 29 September, and 29 October in 2010 for construction of cDNA libraries and on 24 March, 27 April, 22 June, 22 August, and 7 October in 2011 for cDNA microarrays and for anatomical observation.

### Anatomical observation of the cambial zone and the differentiating xylem

Small blocks were collected from stems corresponding to those used for microarray analysis. Thin sections were prepared from embedded tissue in blocks of LR White Resin (London Resin Co., Basingstoke, UK) and stained with safranin and Alcian blue 8GX. Anatomical observations were carried out under both an ordinary light microscope and a polarizing light microscope. The number of cells in the cambial zone and the number of expanding tracheids, secondary wall forming tracheids and lignified tracheids in each radial file were counted under the microscope. The number of cells at each growth stage was statistically compared by a Student’s *t*-test between samples.

### RNA extraction and pyrosequencing

Total RNA was isolated from tissue of the cambium region and differentiating xylem of plus-trees using an RNeasy Plant Mini kit (Qiagen, Gaithersburg, MD, USA) for Chousui8 samples from ten different dates. The quality of total RNA was assessed by measuring the ratio of absorption at 260 nm and 280 nm via an Agilent Bioanalyzer 2100 (Agilent Technologies, Palo Alto, CA, USA). cDNA synthesis from a mixture of ten RNA samples, nebulization, adaptor ligation, emulsion PCR and sequencing were done at Hokkaido System Science Co., Ltd. (Sapporo, Hokkaido, Japan). Sequencing was performed using a Roche 454 Genome Sequencer platform (Roche/454 Life Sciences, Branford, CT, USA) with FLX or Titanium technology.

### Assembly of ESTs from sequences obtained on the 454 platform

Using GS FLX pyrosequencing software, we selected high-quality sequences (> 99.0% accuracy on single base reads) for further processing and assembly. Trimmed and cleaned sequences were assembled using the cDNA assembly feature of Roche Newbler software v. 2.3 (Roche/454 Life Sciences). To obtain clean ESTs, adapter trimming and poly(A/T) removal were performed by the cutadapt tool [113], then short sequences (< 50 bp) were removed and the remaining sequences evaluated using the BLASTN algorithm against *C. japonica* microsatellite sequences obtained from NCBI (http://www.ncbi.nlm.nih.gov) [114], and *Arabidopsis thaliana* retrotransposon sequences obtained from TAIR (http://www.arabidopsis.org) [115]; reads with alignment length of 20 nt or more and percent identity of 90% or more were considered “hit reads” against these sequences. *De novo* assembly was performed using GS De Novo Assembler v2.3 (provided with the Roche GS FLX sequencer) with default parameters (minimum overlap length of 40, minimum percent identity of 90).

### Functional annotation with the BLAST program

The assembled unique sequences putatively encoding proteins were searched against the Arabidopsis protein database in TAIR [115] and the NCBI non-redundant database [114] using the BLASTx algorithm. In addition, our transcripts were also searched against the ForestGEN database [22] using the tBLASTx algorithm. A typical cutoff *E*-value < 1e-5 was used. To identify known protein families, the unique sequences were also searched for the presence of Pfam domain sequences (release 21.0) using the blastx algorithm (*E*-value < 1e-10) [23]. Similarities to ESTs from libraries derived form xylem and/or cambium of *Pinus, Picea*, *Poplar* and Japanese cedar were determined with the tBLASTx program. We used Pinus Gene Index release 6.0 (PGI_libraries PHJ, PHM, ONA, PJD, ERF, 2NV, CJQ, 11 F, 0TU, PJQ, M7S, M7N, 9UQ, 72B, 0U0, 0TT-2, M7Q, M7R, M7P, M7O, PJT, PJM, ERE, CER, PHN, NIL, ERD, BTR, CCS, CJP, 8FB, PJR, ONB, OI1, CJS, ERB, 9NV, 5BN, 1RR, and 0TV), Spruce Gene Index release 2.0 (Sgi_libraries KH2, H5M, H5L, FKG, KGV, EOT, PHL, EOR, KH1, KH0, FH7, FKM, F7N, LCC, F7O, F7U, LCF, IQE, EOS, LCD, LCN, LCM, IQG, IQD, FH9, F7V, IQF, FKL, EOQ, and LCE), Poplar Gene Index release 3.0 (PplGI_libraries EA1, 9BN, BMF, G26, NIQ, EA2, ASV, NL3, FKA, DRG, F8V, F8D, 1CV, LRS, G22, G21, DRC, LRR, DRF, G27, and DQP) from The Gene Index Project website (http://compbio.dfci.harvard.edu/tgi/plant.html) [116], and the ForestGen database (inner bark and sapwood data) as EST databases [22].

PlnTFDB [30], a recently developed database of transcription factor families for 22 plant species, was used to identify putative transcription factors expressed during Japanese cedar wood formation. Blastx searches were performed on matches against *A. thaliana* and *P. trichocarpa* in the PlantTFDB with *E*-values < 1e-5.

The unique sequences were searched locally against a database of clusters of orthologous groups (COGs) from seven eukaryotic genomes [25]. The COGs are comprised of three databases containing orthologous proteins from at least three out of seven eukaryotic species (KOGs), proteins from two species (TWOGs), and lineage-specific expansion groups (LSEs). Sequences with *E*-values < 1e-5 were considered to have significant homology, and were classified following the KOG functional classification.

The sequences of ESTs have been submitted to the DNA Data Bank of Japan under accession numbers DC882454 through DC883482.

### Microarray analysis

We built a custom microarray platform containing 60-mer oligonucleotide probes designed based on 14,612 isotigs (probes to 4 isotigs could be not designed) from all isotigsin proprietary NGS data and 3,470 EST sequences from the “sapwood” and “inner bark” categories (including a full-length cDNA library) in the ForestGen database [22]. A set of 18,082 probes was selected and accommodated in the NimbleGen 4 × 72 K array format (Roche-Nimblegen Inc., Waldkraiburg, Germany), which can examine the expression levels of up to 20,000 genes for four samples at the same time. Therefore, in this format, 18,082 probes were accommodated at least in triplicate in our custom array. For microarray analysis of five sampling dates, we used four biological replicates and three technical replicates for each sample (Additional file 1: Figure S1). Total RNA was extracted with a Plant RNeasy Mini Kit, and DNase was treated in-column with an RNase-Free DNase set (Qiagen). The A_260_/A_280_ ratios of RNA samples used for hybridization ranged from 1.7 to 2.0. An Agilent 2100 Bioanalyzer analyzed the integrity of RNA samples. RNA integrity values of samples used for hybridization ranged from 8.1 to 10.0. Double-stranded cDNA was synthesized using a SuperScript double-stranded cDNA synthesis kit (Invitrogen, Carlsbad, CA, USA) with random 6-mers following the manufacturer’s protocol. Cy3 labeling and hybridization were performed by NimbleGen using standard procedures. Labeled and hybridized slides were scanned using a NimbleGen MS 200 microarray scanner to generate paired files.

Because there were three or four spots for each target, the paired files contained redundant signal intensities for all probes. We took medians as representing intensities to avoid the effect of outliers, and loaded them into Subio platform software (Subio Inc., http://www.subio.jp) [117]. Intensity values were normalized at the 75th percentile, and then transformed into log_2_ ratios based on the average of the 60 samples, which were composed of 5 time points with 12 replicates each. The data presented in this study have been deposited in NCBI’s Gene Expression Omnibus and are accessible through GEO Series access number GSE53034.

Of the total 18,082 target genes, 748 with raw signal intensities not exceeding 1,000 in any samples were filtered out. We calculated the averages of log_2_ ratios at each time point, and excluded an additional 6,273 genes with expression levels hardly varying over time (between −0.5 and 0.5). We tested the 11,061 genes by ANOVA (*p* < 0.05 and BH-FDR < 0.2) to extract 10,380 genes with expression levels that varied for at least one time point. Hierarchical clustering (unweighted pair group method with arithmetic mean, Pearson correlation) was used to identify groups of co-expressed genes. We extracted clusters from tree nodes (Figure 5). We additionally created trees with gene sets manually selected based on biological knowledge.

### Validation of quantitative RT-PCR

Independent verification of microarray results was carried out by qRT-PCR analysis using total RNA from the cambium region tissues used for microarray experiments. Total RNA (500 ng) was reverse-transcribed using the PrimeScript II 1st strand cDNA synthesis kit (Takara Bio, Otsu, Shiga, Japan) with random 6-mers following the manufacturer’s instructions. The resulting first-strand cDNA was diluted 1:5 in water before real-time PCR. Primers were designed using Primer Express software ver. 3.0 (Applied Biosystems, Foster City, CA, USA), with a melting temperature (Tm) between 60 and 65°C, and produced amplicons between 100 and 250 bp. Specific primer pairs were designed for each gene: *Phenylalanine ammonia-lyase* (*PAL*) (isotig 10873: forward 5′-GACCCAGGACGGGAAAGAG-3′, reverse 5′-TAGGCTGGAGTTCAAACGGTTT-3′); *4-Coumarate:CoA ligase* (*4CL*) (isotig 04988: forward 5′-CAGTCGTCGCCAACTATGACA-3′, reverse 5′-ACGGCATCTTCCAGGTCCTT-3′) *Cinnamate-4-hydroxylase* (*C4H*) (isotig 09462: forward 5′-CGTTGAGAAGCTGCCGTATCT-3′, reverse 5′-CGTCAAGGGAGGCTTCTTCA-3′); *Hydroxycinnamoyl-CoA shikimate/quinate hydroxycinnamoyl transferase* (*HCT*) (isotig 10178: forward 5′-GCCCATCCATGATGCAGATT-3′, reverse 5′-GACTGGGCAAAATGAAACCAA-3′); *p-Coumarate-3-hydroxylase* (*C3H*) (isotig 02271: forward 5′-TCACATGGACCCCTCCTGAA-3′, reverse 5′-CGGTAGAGATGCTCAGGCAAT-3′); *Caffeoyl-CoA O-methyltransferase; Cinnamyl alcohol dehydrogenase* (*CCoAOMT*) (isotig 10196: forward 5′-ACTGCAGAGGCTTCCAAGGA-3′, reverse 5′-TCGCTCTGAAGGAGACTCTTGTG-3′); *Cinnamoyl-CoA reductase* (*CCR*) (isotig 05079: forward 5′-CAGGAGCGGGAGGATTTATTG-3′, reverse 5′-CCTCTGGATTGCGAACTGTTC-3′); *Cinnamyl alcohol dehydrogenase* (*CAD*)(isotig 02638: forward 5′-GCAGAGGCAGGCAAGAGATG-3′, reverse 5′-AGTCACATGATGCCCAAATGC-3′); *Cellulose synthase* (*Ces*) (isotig 08498: forward 5′-CATGGCCTGGGAACAACACT-3′, reverse 5′-ATGCGAGGCAGTTCGTTACC-3′); *Sucrose synthase* (*Sus*) (isotig 12351: forward 5′-ACGACTGTTCTTGGCAAACCAT-3′, reverse 5′-ATTGAGCGACCGGAACAAAC-3′); α-*Tubulin* (*Tub α*) (isotig 02753: forward 5′-CATCCTTGGGCACAACATCTC-3′, reverse 5′-TGCCTTTGAGCCTTCTTCCAT-3′); *βTubulin* (*Tub β*) (isotig 13384: forward 5′-TACACTGGTGAGGGCATGGA-3′, reverse 5′-GCATCCTCATCCGCAGTTG-3′); and the endogenous control *Ubiquitin* (*UBQ*) (forward 5′-CGTTAAAGCCAAGATCCAGGACAA-3′, reverse 5′-TCCATCCTCAAGCTGTTTCCCA-3′)*.* For each sample, triplicate quantitative PCR assays were performed using Power SYBR Green PCR master mix (Applied Biosystems) with ROX reference dye according to the manufacturer’s protocol. Amplification was carried out with a StepOnePlus system (Applied Biosystems). After an initial 10-min activation step at 95°C, 40 cycles (95°C for 15 s and 60°C for 1 min) were performed, and a single fluorescent reading was obtained after each cycle immediately following the annealing/elongation step at 60°C. Preliminary quantitative PCR assays were performed to evaluate primer pair efficiency and absence of genomic DNA contamination using a negative control. A melting curve analysis was performed at the end of cycling to ensure amplification of a single product. For relative quantification and comparisons, we used the delta-delta-Ct method with *Ubiquitin* as the normalization internal control gene.

## Competing interests

The authors declare that they have no competing interests.

## Authors’ contributions

KM carried out construction of the cDNA library and microarray experiments, qRT-PCR validation, and manuscript preparation. MT conducted part of the analysis of EST data against databases. TF, TI, KK, KY and YF helped sample materials and performed anatomical observations. TF and AW proposed the research project and guided the research process. All authors have read and approved the final version of the manuscript.

## Supplementary Material

Additional file 1: Figure S1. Chart of this study. Click here for file

Additional file 2: **Differentially expressed targets during the growing season.** A set of 10,380 *C. japonica* genes that were differentially expressed. Gene order is the same as in Figure 5 cluster diagrams. Click here for file

Additional file 3: Description and expression profile of the individual targets listed for Figures 6,7,8,9,10,11,12,13,14,15,16,17,18,19, and 20. Click here for file

Additional file 4: Figure S2. Daily maximum and minimum temperatures measured at the sampling site. Sampling days are indicated: 24 March, 27 April, 22 June, 24 August and 7 October, 2011. Click here for file
