# Erratum to: Transcriptome sequencing and profiling of expressed genes in cambial zone and differentiating xylem of Japanese cedar (*Cryptomeria japonica*)

**DOI:** 10.1186/s12864-016-3156-6

**Published:** 2016-10-17

**Authors:** Kentaro Mishima, Takeshi Fujiwara, Taiichi Iki, Katsushi Kuroda, Kana Yamashita, Miho Tamura, Yoshitake Fujisawa, Atsushi Watanabe

**Affiliations:** 1 Forest Tree Breeding Center, Forestry and Forest Products Research Institute, 3809-1 Ishi, Juo, Hitachi, Ibaraki 319-1301 Japan; 2 Forestry and Forest Products Research Institute, 1 Matsunosato, Tsukuba, Ibaraki 305-8687 Japan; 3 Department of Forest Environmental Sciences, Faculty of Agriculture, Kyushu University, 6-10-1 Hakozaki, Higashi-ku, Fukuoka 812-8581 Japan

## Erratum

In the original publication of this article [1], the accession number was listed as DC882454 through DC883482 on page 27; instead, the number should be DRA000525.
