# Supplementary material for: Transcriptome sequencing and profiling of expressed genes in cambial zone and differentiating xylem of Japanese cedar (Cryptomeria japonica)
Source: BMC Genomics. 2014 Mar 20;15:219. doi: 10.1186/1471-2164-15-219 (PMC3999911; doi:10.1186/1471-2164-15-219)
Supplement: Additional file 1: Figure S1. — Chart of this study. [file 1471-2164-15-219-S1.pdf]

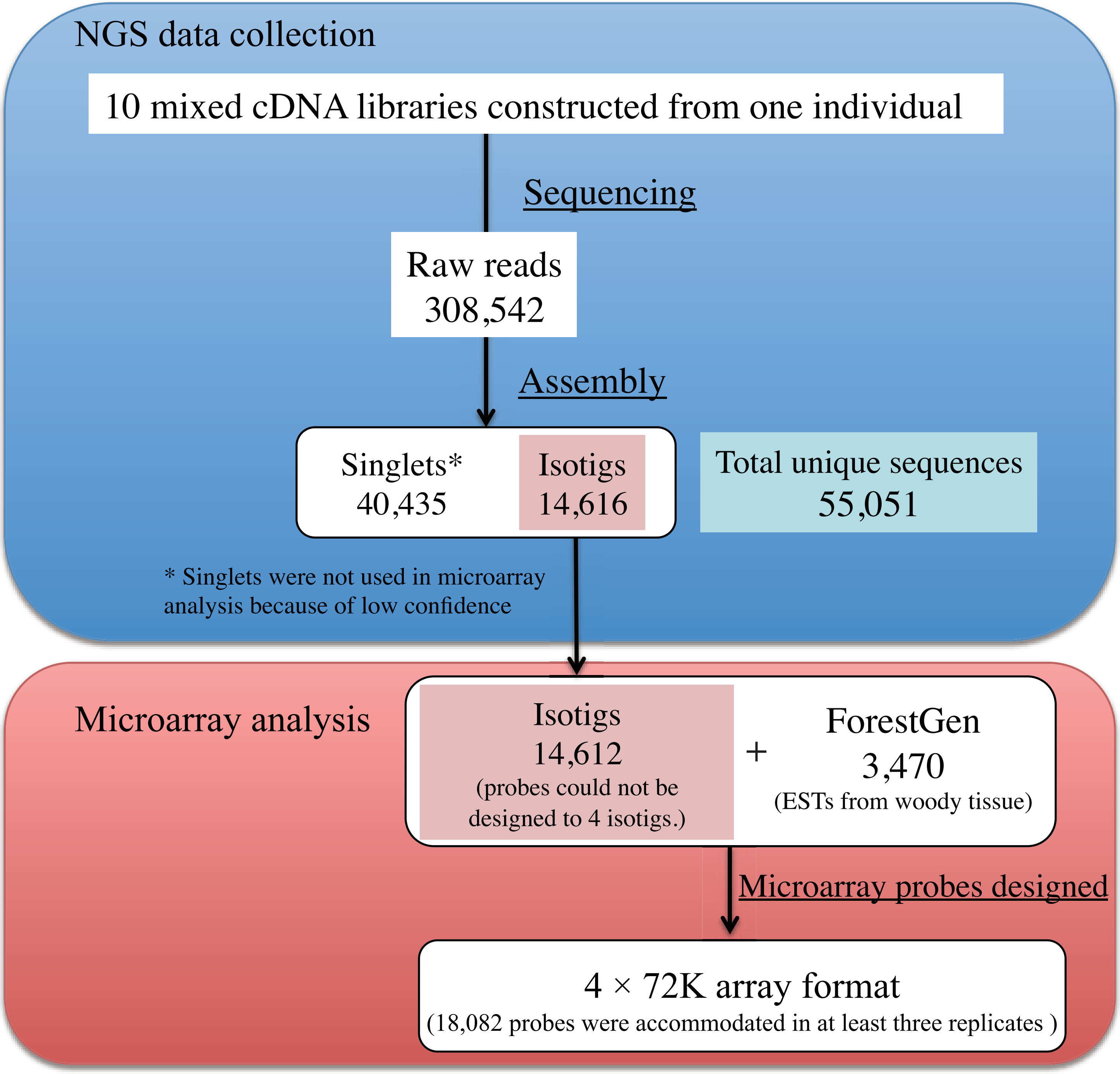
NGS data collection
10 mixed cDNA libraries constructed from one individual
Sequencing
Raw reads
308,542
Assembly
Singlets*
40,435
Isotigs
14,616
Total unique sequences
55,051
* Singlets were not used in microarray analysis because of low confidence
Microarray analysis
Isotigs
14,612
(probes could not be designed to 4 isotigs.)
+
ForestGen
3,470
(ESTs from woody tissue)
Microarray probes designed
4 × 72K array format
(18,082 probes were accommodated in at least three replicates)
