# Supplementary figures and images for: Transcriptome sequencing and profiling of expressed genes in cambial zone and differentiating xylem of Japanese cedar (Cryptomeria japonica)
Source: BMC Genomics. 2014 Mar 20;15:219. doi: 10.1186/1471-2164-15-219 (PMC3999911; doi:10.1186/1471-2164-15-219)

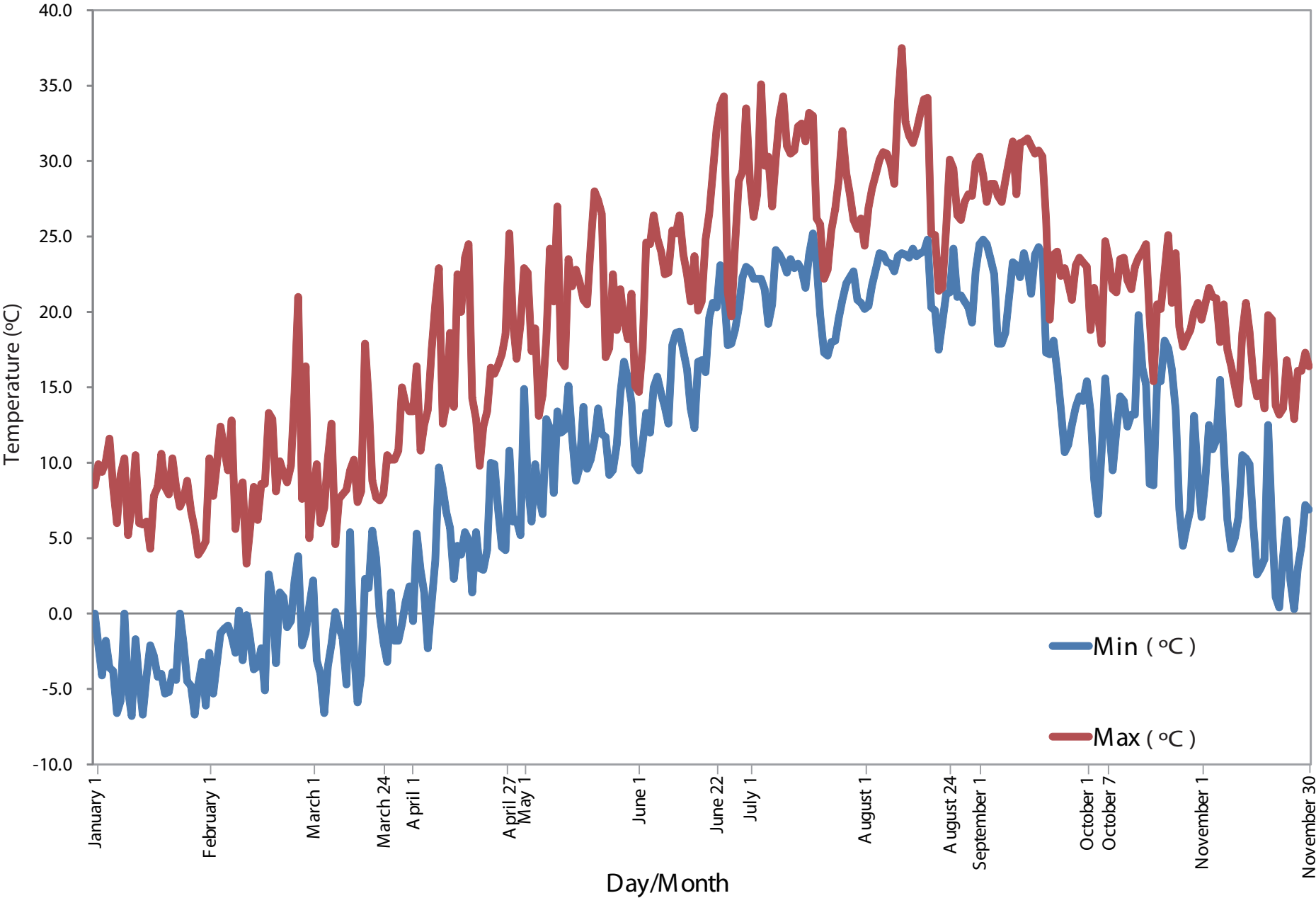

Supplement: Additional file 4: Figure S2. — Daily maximum and minimum temperatures measured at the sampling site. Sampling days are indicated: 24 March, 27 April, 22 June, 24 August and 7 October, 2011. [file 1471-2164-15-219-S4.pdf]
